# Hijacking Sodium–Glucose Cotransporters: Fructose Drives Neuronal and Microglial Dysfunction

**DOI:** 10.1080/17590914.2026.2696821

**Published:** 2026-07-10

**Authors:** Salaheldeen Elsaid, Muhammad Asad Usmani, Xiangdong Wu, Junkai Hu, Nigus Ambaye, Sui Seng Tee

**Affiliations:** Department of Radiology, School of Medicine, University of Maryland, Baltimore, Maryland, USA

**Keywords:** Fructose uptake, hippocampal neurons, microglia, neuroinflammation, SGLTs

## Abstract

Excess fructose consumption has been implicated in metabolic disease, yet its impact on brain physiology and cellular metabolism remains poorly understood. The hippocampus expresses fructose transporters and fructolytic enzymes, suggesting potential vulnerability to fructose-induced metabolic stress. Here, we investigated fructose uptake mechanisms and downstream functional responses in BV2 microglia and HT22 hippocampal neurons using sodium manipulation, pharmacological inhibition, transporter expression profiling, and live-cell fluorescent sugar uptake assays. Hippocampal neurons exhibited strong sodium-sensitive, phlorizin-responsive fructose uptake, accompanied by reduced expression of facilitated hexose transporters and selective induction of Sglt1. In contrast, microglia demonstrated both sodium-sensitive and sodium-independent components of fructose uptake, associated with coordinated remodeling of GLUT and SGLT family members. Functionally, fructose exposure was associated with membrane hyperpolarization and reduced extracellular vesicle (EV) release in neurons, whereas microglia displayed membrane depolarization, enhanced EV secretion, and induction of pro-inflammatory genes. Knockdown of ketohexokinase (KHK) attenuated inflammatory gene expression and EV release in microglia, while pharmacological inhibition of sodium-dependent transport selectively reduced EV secretion. Complementary secondary analysis of hippocampal RNA-seq data from mice exposed to high-fat/high-fructose feeding revealed coordinated regulation of sodium-coupled transporters, ion channels, and synaptic gene programs, with partial normalization following SGLT inhibition. Together, these findings identify cell-type-specific fructose handling strategies in hippocampal neurons and microglia and suggest that sodium-dependent transport and fructolytic metabolism differentially influence membrane polarization, vesicle signaling, and inflammatory activation under metabolic stress.

## Introduction

The consumption of fructose-containing sweeteners and high-fat foods has increased over recent decades (Du et al., 2025; Hassel et al., 2015; 2015; Jensen et al., 2018; 2018; Krause & Wegner, 2020; Ting, 2024) and has been associated with metabolic dysfunction and alterations in brain metabolism (Sturno et al., 2025; Yaribeygi et al., 2024). Chronic high-fat feeding is associated with systemic insulin resistance, neuroinflammation, oxidative stress, and cognitive impairment, with the hippocampus emerging as a particularly vulnerable brain region (Du et al., 2025; Jiang et al., 2025; Zhang et al., 2025). Western dietary patterns rarely involve excessive fat consumption in isolation and frequently combine high-fat intake with fructose-containing sweeteners (Clemente-Suárez et al., 2023). Fructose differs from glucose in that it elicits minimal insulin release and satiety signaling, thereby promoting chronic metabolic stress (DiStefano & Shaibi, 2021). Beyond its well-established systemic effects (Jensen et al., 2018; Mirtschink et al., 2018), accumulating evidence suggests that fructose influences central nervous system metabolism, with particular vulnerability of the hippocampus, a region essential for learning and memory (Celikbilek et al., 2018; Zhang et al., 2025). These observations provide the rationale for investigating how fructose contributes to cellular dysfunction within the context of diet-induced metabolic stress.

Multiple brain regions, including the hippocampus, cortex, hypothalamus, and cerebellum, express core components of fructose metabolism, including the fructose transporter GLUT5 (SLC2A5) and ketohexokinase (KHK) (Iizuka, 2023; Li et al., 2023; Ting, 2024). Consistent with this, functional fructose utilization has been demonstrated in ex vivo brain preparations and in vivo tracer studies (Izumi & Zorumski, 2009). Cerebral fructose exposure is not solely diet-dependent; under hyperglycemic conditions, up to 30% of excess glucose is converted to fructose via the polyol pathway, leading to sustained endogenous fructose accumulation in the cerebrospinal fluid and hippocampus during metabolic disease (Hwang et al., 2017; Spinowitz & Altszuler, 1973; Tigchelaar et al., 2022).

Microglia, the brain’s resident immune cells are particularly sensitive to fructose. They express high levels of GLUT5 and KHK that are further upregulated during aging and neurodegeneration (Li et al., 2023; Wang et al., 2025). Unlike glycolysis, fructose metabolism in microglia proceeds without the feedback regulation leading to rapid ATP depletion, AMP accumulation, uric acid generation, and excessive reactive oxygen species (ROS) production (Li et al., 2023; Wang et al., 2025). This metabolic stress drives a pro-inflammatory phenotype marked by chronic cytokine release (Cigliano et al., 2018; Shen et al., 2025). Fructose exposure has been shown to activate microglial inflammatory pathways both in vitro and in vivo (Shen et al., 2025; Wang et al., 2025), suggesting that microglial fructose metabolism may contribute to neuroinflammation and synaptic vulnerability (Li et al., 2023; Sturno et al., 2025).

Hippocampal neurons also exhibit sensitivity to fructose exposure. Fructose feeding increases uric acid accumulation, and activation of inflammatory signaling pathways, induces mitochondrial dysfunction, and reduces synaptic integrity markers including Dlg4 (Cigliano et al., 2018; Mazzoli et al., 2021). In neuronal models, fructose suppresses synaptic and neurotrophic gene programs while increasing oxidative burden, effects partially reversible upon fructose withdrawal (Cigliano et al., 2018; Jiménez-Maldonado et al., 2018). Together, these findings indicate that fructose exposure may influence both neuronal signaling and neuroimmune interactions within the hippocampus.

Despite the dominant focus on the GLUT5–KHK axis in cerebral fructose handling, the contribution of sodium-glucose cotransporters (SGLTs) to brain fructose handling remains incompletely understood. Members of the SGLT family are expressed in hippocampal neurons, microglial cells, and choroid plexus epithelial cells contributing to cerebrospinal fluid nutrient homeostasis (Yu et al., 2010; 2013). Given that SGLT inhibitors are clinically approved and exhibit emerging neuroprotective effects (Rizzo et al., 2022; Yaribeygi et al., 2024), their role in modulating brain fructose uptake represents a critical knowledge gap with significant translational relevance.

Here, we define cell-type–specific fructose uptake pathways in BV2 microglia and HT22 hippocampal neurons, integrating sodium-dependent and sodium-independent transport with downstream metabolic and inflammatory outcomes. By defining distinct fructose-handling strategies across hippocampal cell types, this study provides a framework for understanding how metabolic stress may differentially influence neuronal and microglial signaling.

## Materials and Methods

### Cell Culture and Differentiation

BV-2 cells are a female-derived murine microglial line. HT22 cells are a mouse hippocampal neuronal cell line; however, the gender of the donor could not be confirmed from the available cell-line documentation. Both cells were maintained in Dulbecco’s Modified Eagle Medium (DMEM; high glucose, 25 mM) supplemented with 10% fetal bovine serum and 1% penicillin–streptomycin at 37 °C in a humidified incubator with 5% CO_2_.

For neuronal differentiation, HT22 cells were transitioned to a modified serum-free differentiation medium based on DMEM supplemented with N2 nutrient supplement (1x), INV-21103049, nerve growth factor (NGF; 50 ng/mL for induction, SIG-N5415 reduced to 5 ng/mL during experimental treatments), phorbol 12,13-dibutyrate (100 nM), MCE-HY-18985, and dibutyryl cyclic AMP (100 μM), MCE-HY-B0764. Cells were initially expanded in serum-containing DMEM and subsequently switched to differentiation medium for 24–48 h prior to experimental manipulation.

For all sugar uptake, membrane potential, and extracellular vesicle (EV) assays, cells were plated to achieve approximately 70%–80% confluence and serum-starved for 2 h before treatment in 5 mM of glucose or fructose.

Human induced pluripotent stem cell (iPSC, healthy control cells, Ctrl^WT/WT^, ID ND34791) were generously provided by Dr. Awad and were differentiated using established protocols (Momcilovic et al., 2016). Neuronal differentiation was further confirmed by immunofluorescence staining for the neuronal marker βIII-tubulin (TUJ1), demonstrating robust neuronal morphology and neurite outgrowth (Supplementary Fig. 5), in addition to previously validated by expression of hippocampal neuronal markers, including GRIN1(Awad et al., 2015).

### Sugar Uptake Assays

Glucose and fructose uptake were assessed using 100 µM of the fluorescent hexose analogs 2-NBDG (for glucose) and NBD-fructose. Cells were incubated in Krebs–Ringer–HEPES (KRH) buffer containing sodium (Na^+^-KRH), 50 mM HEPES, 137 mM NaCl, 4.7 mMKCl, 1.85 mM CaCl2, and 1.3 mM MgSO4, 0.1%(w/v) bovine serum albumin (BSA; e.g., Wako Chemicals, cat. no. 011–07493) or in sodium-free KRH buffer in which NaCl was replaced with equimolar 137 mM N-methyl-D-glucamine (NMDG; Sigma, M2004) to evaluate sodium-dependent transport.

Where indicated, cells were pretreated for 2 hours with 0.5 mM AICAR (AMPK activator; MCE-HY-13417) and/or 0.1 mM phlorizin (sodium-glucose cotransporter inhibitor; MCE-HY-N0143) prior to initiation of uptake assays. Pretreatment was performed in KRH buffer under the same sodium conditions used for uptake measurements.

Cells were seeded in black-walled, clear-bottom 96-well plates. Fluorescent hexoses were added at a final concentration of 100 µM (NBD-glucose, MCE-HY-116215; NBD-fructose, Cayman 9002314). Uptake was monitored over a 60-minute time course at 5-minute intervals to define the linear phase of substrate accumulation. Based on preliminary kinetic analyses, a single time point within the linear range was selected for quantitative comparisons (15 minutes for glucose; 40 minutes for fructose). Uptake reactions were terminated by rapid washing with ice-cold KRH buffer to remove extracellular substrate. Fluorescence intensity was measured using a TECAN Spark microplate reader. Background fluorescence was determined in parallel wells containing cells incubated under identical conditions without fluorescent substrate and was subtracted from all experimental readings prior to normalization. Values were normalized to their respective control conditions and expressed as percentage of control for statistical analysis.

### Membrane Potential Measurements

Changes in membrane potential were assessed using the voltage-sensitive dye DiBAC4 (3) (MCE-HY-101892). Cells were kept in glucose or fructose at 5 mM final concentration and preincubated with DiBAC4 (3) (2 μM), for 30 min at 37 °C then for another 1 hr under indicated treatment conditions. Increased fluorescence reflects membrane depolarization, whereas reduced fluorescence indicates hyperpolarization. Fluorescence intensity was measured using a TECAN Spark microplate reader at excitation/emission: 490/516 nm. Values were normalized to their respective confluency conditions and expressed as percentage of control for statistical analysis.

### Extracellular Vesicle Isolation and Quantification

EVs were isolated from conditioned media using differential centrifugation. Media were sequentially centrifuged at 300 × g (10 min), 2,000 × g (20 min), and 10,000 × g (30 min) (Zhang et al., 2023). Extracellular vesicles were characterized using nanoparticle tracking analysis (NTA) on a NanoSight NS300 instrument (Malvern Panalytical, Malvern, UK). For each sample, three independent recordings were acquired and analyzed using NanoSight software according to the manufacturer’s recommendations. Particle concentration (particles/mL) and size-distribution profiles were determined from the averaged recordings. Representative particle size-distribution profiles and EV concentration measurements are shown in Supplementary Figures S4. Data are presented as mean ± SD from three independent biological replicates. EV protein content was assessed by immunoblotting for CD9 and IL-6.

### KHK Knockdown

For some BV2 cells, Lipofectamine RNAiMAX (INV-13778030) was used to transfect either 100 pmol scramble (Invitrogen, 465370) siRNA or stealth siRNA targeting Khk to knock it down (Invitrogen, KHKHSS105797).

### Gene Expression Analysis

Total RNA was extracted using Purelink RNA Mini Kit (Invitrogen, 12183018 A) and reverse-transcribed using High-capacity RNA-to-cDNA Kit (Invitrogen 4387406) according to the manufacturer’s instructions. Quantitative real-time PCR was performed using SYBR select (Invitrogen 4472908) on QuantStudio 5 (Applied Biosystems). All primers, Table 1 is specifically designed for this study and were purchased from IDT.

**Table 1.** Primer sequences used in qPCR.

Mouse primers		
*Khk*	GGATGTGTCTCAAGTGACTTGGC	GTCCTTAGCAGACACATCTGGC
*Grin1*	CCTTTCAGAGCACACTGTGGCT	CCAGGAAAACCACATGGCAGAG
*Dlg4*	TCAGACGGTCACGATCATCGCT	GTTGCTTCGCAGAGATGCAGTC
*Kcna1*	GAGTCGCACTTCTCCAGTATCC	CCCACGATCTTGCCTCCAATTG
*Glut1*	GCTTCTCCAACTGGACCTCAAAC	ACGAGGAGCACCGTGAAGATGA
*Glut2*	GTTGGAAGAGGAAGTCAGGGCA	ATCACGGAGACCTTCTGCTCAG
*Glut4*	GGTGTGGTCAATACGGTCTTCAC	AGCAGAGCCACGGTCATCAAGA
*Glut5*	ATCGCTGCCTTTGGCTCATCCT	AGCAGCGTCAAGGTGAAGGACT
*Sglt1*	CTCGTGGTGGAACTCATGCCTA	GCGCTGTTGAAGATGGAGGTCA
*Sglt2*	CAGACCTTCGTCATTCTTGCCG	GTGCTGGAGATGTTGCCAACAG
*Sglt3*	CCTCTTCACCATCGACCTGTAC	CACAGGATGCTGACGGCGATTA
*Sglt4*	GCTGGTTATGGCTCTCATGCCT	CACATCTATGGCAAACAGGGTGC
*Sglt5*	GATTGGCTGCTCCAACATTGCC	CTGCTGTTGAAGATGGAGGTCAG
*Actin*	CATTGCTGACAGGATGCAGAAGG	TGCTGGAAGGTGGACAGTGAGG
Human primers		
*Grin1*	CCTTTCAGAGCACACTGTGGCT	CCAGGAAAACCACATGGCAGAG
*Syp*	TCGGCTTTGTGAAGGTGCTGCA	TCACTCTCGGTCTTGTTGGCAC
*Kcna1*	CATCTTTTGCCTGGAGACGCTC	GGAGAACCAGATGATACACAGCG
*Actin*	CACCATTGGCAATGAGCGGTTC	AGGTCTTTGCGGATGTCCACGT

### RNA-Seq Analysis of Hippocampal Tissue

To provide transcriptomic context for functional pathways in the brain, we performed a secondary analysis of a publicly available hippocampal RNA-seq dataset (GSE261887) generated from mice exposed to a high-fat diet supplemented with 10% fructose in drinking water (Zhang et al., 2025). In the original study, mice were assigned to three groups: control mice maintained on standard chow (AIN-93 M, Trophic Animal Feed Hightech Co. Ltd, Nantong, China) and water. and tap water, mice fed a high-fat diet (TP 23,100; 45% kcal derived from fat) supplemented with 10% fructose in drinking water (HFFD), and HFFD mice treated with the sodium–glucose cotransporter inhibitor phlorizin (HFFD+Ph).

RNA-seq analysis was performed using iDEP version 2.01 (Ge et al., 2018). Raw count matrices obtained from GSE261887 were uploaded into iDEP, and lowly expressed genes were filtered using the platform’s default settings. Gene expression data were normalized using the DESeq2 median-of-ratios method to account for differences in sequencing depth and library composition among samples. Differential expression analysis was performed using DESeq2, and adjusted *p*-values were calculated using the Benjamini–Hochberg false discovery rate (FDR) correction. Genes with an adjusted *p*-value (FDR) < 0.05 were considered significantly differentially expressed. Normalized expression values were used for pathway enrichment analyses, volcano plots, and gene expression visualizations.

For graphical presentation of selected genes, normalized expression values are shown as mean ± SD. Statistical comparisons among groups were performed using one-way ANOVA followed by Tukey’s multiple-comparisons test using GraphPad Prism version 9 (GraphPad Software, San Diego, CA, USA). A *p*-value < 0.05 was considered statistically significant.

#### Western Blotting

Extracellular vesicles (EVs) were isolated from conditioned cell culture media as described above. EV protein lysates were prepared in Laemmli buffer. Equal amounts of EV protein (50 µg per lane) were resolved by SDS-PAGE using Sure-PAGE gels (GenScript, M00652) and transferred onto PVDF membranes (Invitrogen, LC2005). Membranes were blocked in 5% non-fat dry milk in TBST for 1 hour at room temperature and incubated overnight at 4 °C with primary antibodies diluted 1:1000 in 3% BSA in TBST. The following primary antibodies were used: anti-IL-6 (mouse monoclonal, Invitrogen, Cat# M620, RRID: AB_223576) and anti-CD9 (rabbit monoclonal, Cell Signaling Technology, Cat# 13174, RRID: AB_2798139). After washing, membranes were incubated with HRP-conjugated secondary antibodies for 1 hour at room temperature. Protein bands were visualized using enhanced chemiluminescence detection. Images were acquired under non-saturating conditions using ChemiDoc MP Imaging System (Catalog #1708280, Bio-Rad Laboratories, Inc.). Only linear adjustments to brightness and contrast were applied uniformly across the entire image.

### Statistical Analysis

All data are presented as mean ± standard deviation (SD). The number of independent biological replicates (*n*) for each experiment is indicated below and in the corresponding figure legends. Technical replicates were averaged prior to statistical analysis.

For RNA sequencing experiments, *n* = 4 animals per group were used. For quantitative PCR (qPCR) analysis, *n* = 3 independent biological replicates were performed for each treatment condition. Sugar uptake experiments were conducted with *n* = 4 independent biological replicates per treatment. Membrane potential assays were performed with *n* = 6 independent biological replicates for BV2 cells and *n* = 5 independent biological replicates for HT22 cells.

Comparisons between two groups were performed using unpaired two-tailed Student’s t-tests. Comparisons among multiple groups were conducted using one-way or two-way analysis of variance (ANOVA), followed by Tukey’s multiple-comparisons test. All statistical analyses were performed using GraphPad Prism (version 9, GraphPad Software, San Diego, CA, USA).

## Results

### Secondary Analysis of Hippocampal Transcriptomic Responses to Combined High-Fat and Fructose Exposure

**Figure 1.**
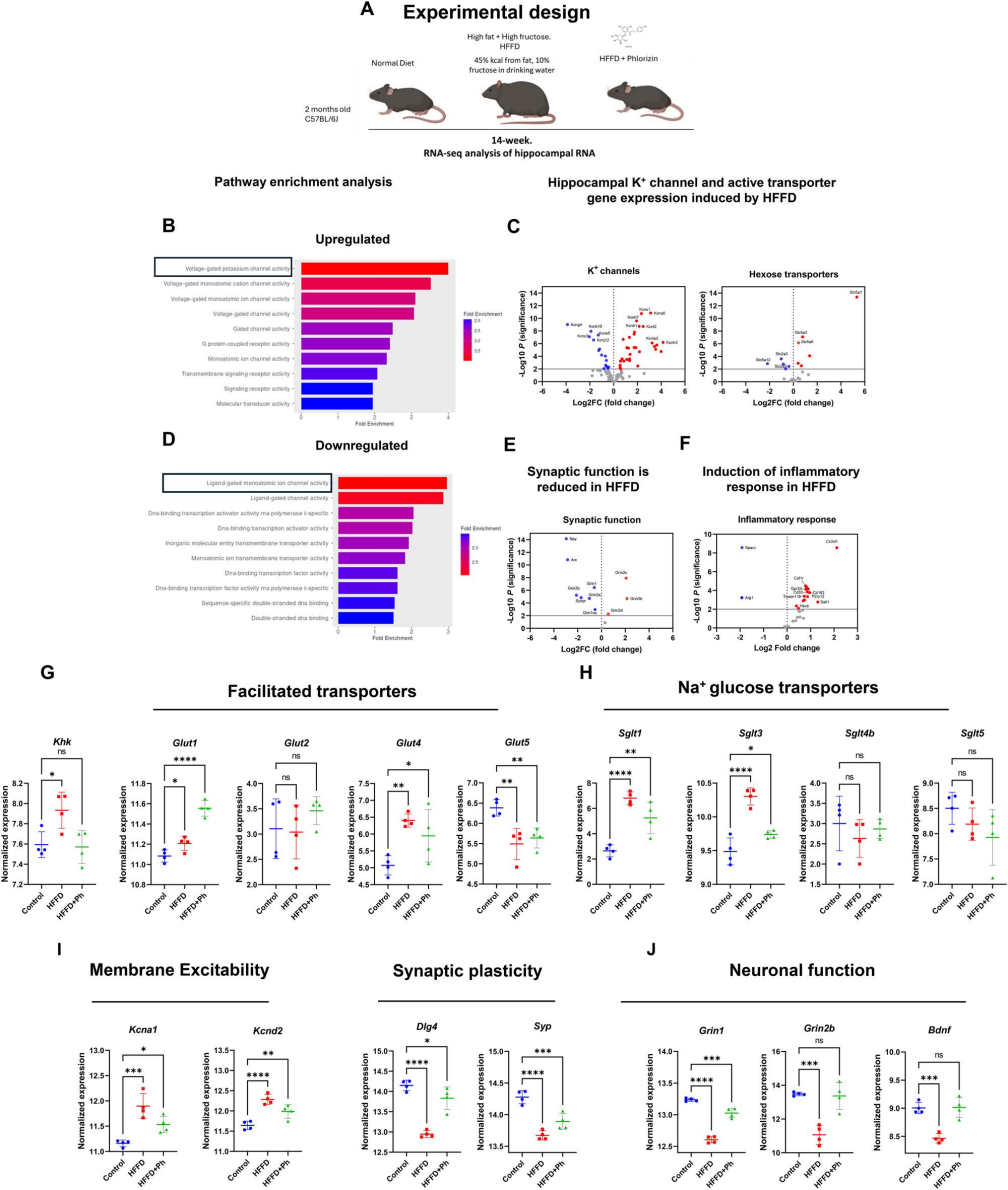
High-fat/high-fructose feeding is associated with coordinated remodeling of hippocampal transporter, ion channel, synaptic, and inflammatory gene programs, with partial normalization following phlorizin treatment. (A) Experimental design. C57BL/6J mice were maintained on control diet or high-fat diet supplemented with 10% fructose in drinking water (HFFD) for 14 weeks. A subset of HFFD mice received phlorizin treatment. Hippocampal RNA was subjected to transcriptomic analysis (GSE261887). (B) Pathway enrichment analysis of hippocampal RNA-seq data comparing HFFD versus control diet. Voltage-gated potassium channel activity and membrane-associated transport pathways were among the most significantly upregulated gene sets. (C) Volcano plots highlighting differential expression of potassium channel genes and sodium-glucose cotransporters in HFFD hippocampi relative to controls. (D) Downregulated pathways in HFFD hippocampi include ligand-gated ion channel activity and synaptic transmission–related processes. (E) Volcano plot of synaptic function–associated genes showing reduced expression in HFFD conditions. (F) Volcano plot of inflammatory response–associated genes showing increased expression in HFFD hippocampi. (G) Expression of Khk and facilitated hexose transporters (Glut family) across control, HFFD, and HFFD + phlorizin groups. (H) Expression of sodium-dependent transporters (Sglt1, Sglt3, Sglt4b, and Sglt5) under the same conditions. (I) Expression of genes associated with membrane excitability (Kcna1 and Kcnd2) and synaptic markers (Dlg4, Syp). (J) Expression of neuronal plasticity–associated genes (Grin1, Grin2b, and Bdnf). Across multiple transporters, ion channel, and synaptic gene categories, HFFD was associated with significant transcriptional remodeling, with partial normalization observed following phlorizin treatment. Data are presented as mean ± SD (*n* = 4 biological replicates per group). Statistical analysis was performed using one-way ANOVA followed by Tukey’s multiple-comparisons test.

To provide transcriptomic context for functional pathways in the brain, we performed secondary analysis of a publicly available hippocampal RNA-seq dataset (GSE261887) generated from mice exposed to TP 23,100 diet, a high-fat formulation providing 45% of total caloric intake from fat, supplemented with 10% fructose in drinking water (Zhang et al., 2025). Animals in the original study were assigned to three groups: control mice fed standard chow with tap water, mice fed high-fat diet plus 10% fructose (HFFD), and HFFD mice treated with the sodium-glucose cotransporter inhibitor phlorizin (HFFD+Ph) (Figure 1A).

#### Diet-Induced Metabolic Stress Alters Hippocampal K^+^ Channels Expression, Synaptic Signaling, and Immune Pathways

Pathway enrichment analysis identified voltage-gated K^+^ channel activity among the most significantly upregulated pathways in HFFD mice, with volcano plot analysis revealing coordinated induction of multiple K^+^ channel subunits (Figure 1B,C). In parallel, sodium-dependent transporters Sglt1 (Slc5a1), Sglt3 (Slc5a3), and Sglt6 (Slc5a6) were upregulated, consistent with potential alterations in sodium-coupled sugar transport capacity under combined metabolic stress.

Conversely, ligand-gated ion channel activity was significantly downregulated, accompanied by reduced expression of genes encoding synaptic receptors and neurotransmission-associated components (Figure 1D and E). Enrichment of immune-related pathways was observed, together with downregulation of the arginine biosynthesis pathway, including reduced expression of Sparc and Arg1 (Figure 1F). Because these data derive from bulk hippocampal tissue, cell-type-specific contributions cannot be resolved, and the observed immune signature cannot be definitively attributed to a particular cellular population. Together, these findings indicate coordinated transcriptional remodeling of ion channel, transporter, synaptic, and immune-associated gene programs in the hippocampus under high-fat/high-fructose dietary conditions.

#### Phlorizin Treatment Partially Rescues Diet-Induced Transcriptional Alterations in Transporters, Ion Channels, and Synaptic Genes in the Hippocampus

Phlorizin treatment in HFFD mice was associated with partial normalization of several transporters, ion channels (Figure 1G and H), synaptic and ion channel genes (Figure 1I and J), including restoration of *Khk*, *Glut4*, *Sglt1*, *Kcna1*, and *Grin1* expression toward control levels.

However, not all transcriptional alterations were fully restored. These findings suggest selective modulation of diet-associated transcriptional programs following pharmacological inhibition of sodium-dependent glucose transport.

To further characterize endogenous sugar transport pathways in the hippocampus, we examined the expression of additional facilitated sugar transporters (GLUTs) and sodium-dependent glucose cotransporters (SGLTs) within the RNA-seq dataset (Supplementary Fig. S3). HFFD induced selective remodeling of multiple transporter families. Among the GLUT transporters examined, GLUT3 expression was significantly reduced whereas GLUT9 expression was significantly increased relative to controls, while GLUT6 and GLUT8 remained unchanged. Analysis of SGLT family members revealed increased expression of SGLT6, SGLT7, SGLT8, and SGLT11 in HFFD mice, suggesting enhanced sodium-dependent sugar transport capacity under metabolic stress. Several of these alterations were partially normalized following phlorizin treatment. Together, these findings indicate that HFFD induces broad transcriptional remodeling of both facilitated and sodium-dependent sugar transport systems in the hippocampus.

Since the dietary paradigm combined high fat and fructose, the specific contribution of fructose alone cannot be isolated from these data. Accordingly, this transcriptomic analysis provides contextual evidence of hippocampal remodeling under combined metabolic stress rather than direct proof of fructose-specific effects.

These results position phlorizin as a modulator of hippocampal gene expression under metabolic stress and underscore the need to move beyond bulk transcriptomics to isolate cell-type-specific responses, which we pursued through in vitro models using HT22 hippocampal neurons and BV2 microglial cells.

### Differential Regulation of Glucose and Fructose Uptake in HT22 Neurons and BV2 Microglia

**Figure 2.**
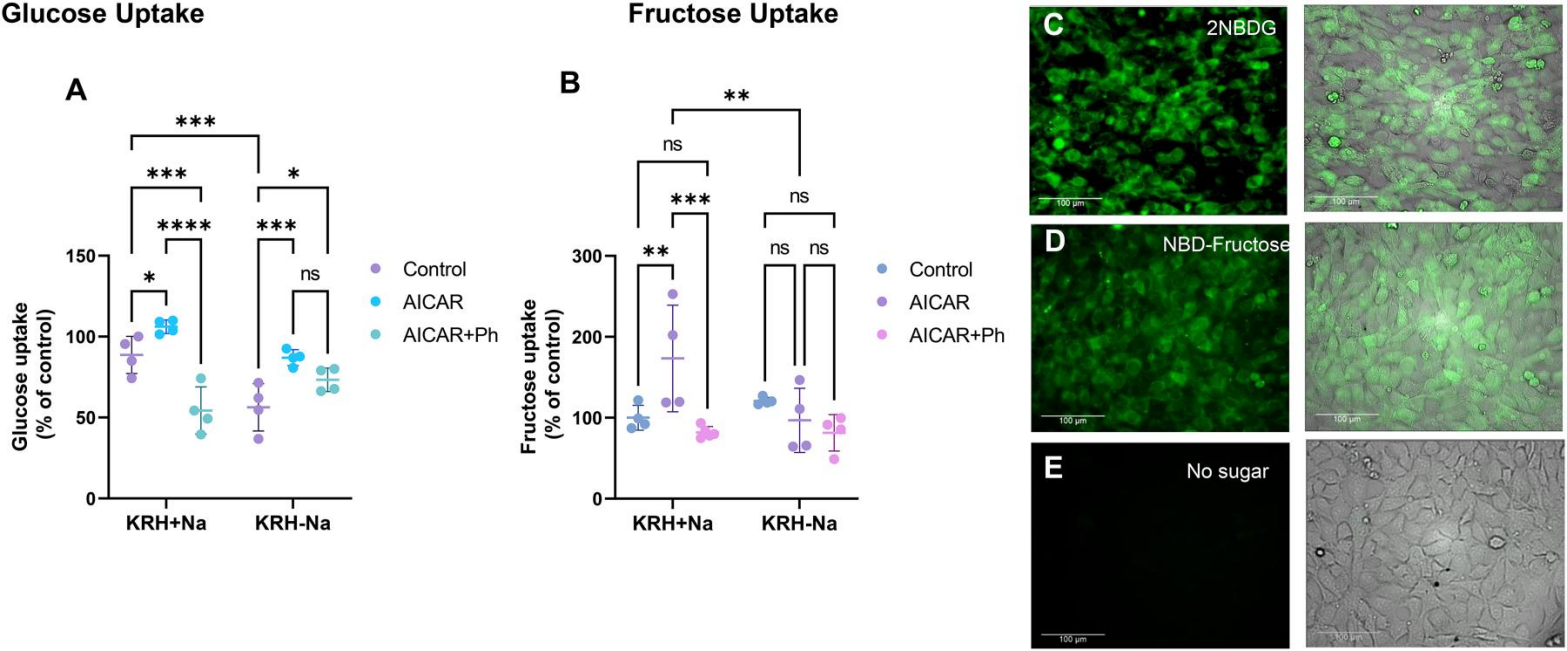
Neurons exhibit sodium-dependent fructose uptake. (A and B) Quantification of glucose uptake (2NBDG) and fructose uptake (NBD-fructose) in HT22 neurons. (C and D) Representative fluorescence images showing uptake of 2NBDG and NBD-fructose. (E) No-sugar control demonstrating minimal background fluorescence. S Data are presented as mean ± SD (*n* = 4 biological replicates per group). Statistical analysis was performed using two-way ANOVA followed by Tukey’s multiple-comparisons test; ns, not significant; *P < 0.05; **P < 0.01; ****P < 0.0001.

**Figure 3.**
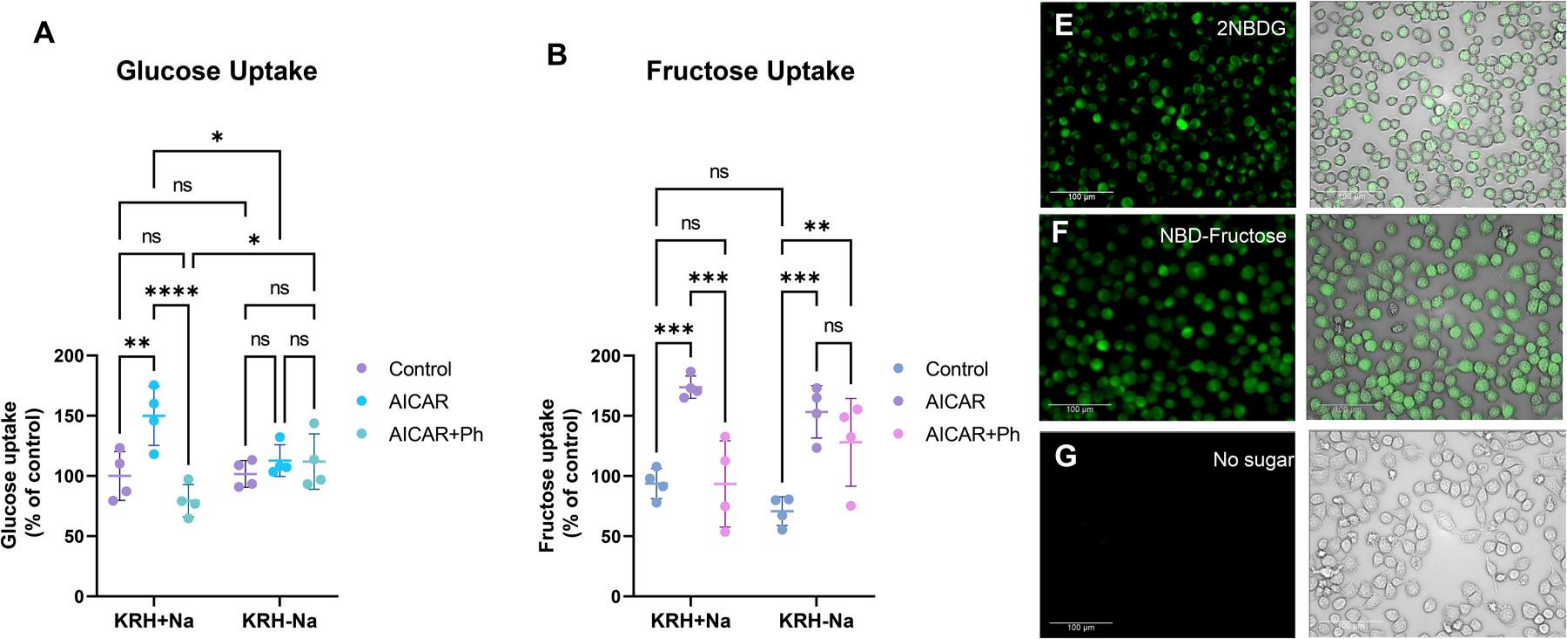
Microglia exhibit sodium-dependent fructose uptake. (A and B) Quantification of glucose uptake (2NBDG) and fructose uptake (NBD-fructose) in BV2 microglia. (C and D) Representative fluorescence images showing uptake of 2NBDG and NBD-fructose. (E) No-sugar control demonstrating minimal background fluorescence. Data are presented as mean ± SD (*n* = 4 biological replicates per group). Statistical analysis was performed using two-way ANOVA followed by Tukey’s multiple-comparisons test. ns, not significant; *P < 0.05; **P < 0.01*, P* < 0.01; ***P < 0.001; ****P < 0.0001.

To compare glucose and fructose uptake under defined experimental conditions, uptake kinetics were first assessed over 60 minutes to identify the linear phase of substrate accumulation (data not shown). Based on these preliminary analyses, quantitative comparisons were performed at 15 minutes for glucose and 40 minutes for fructose (Figures 2C–E and 3C–E). Fluorescence values were background-subtracted, normalized to control conditions, and expressed as percentage of control.

To probe regulatory and sodium-sensitive components of hexose transport, cells were treated with the AMPK activator AICAR and the sodium-glucose cotransporter inhibitor phlorizin under sodium-containing (KRH+Na) or sodium-free (KRH − Na) conditions.

#### HT22 Neurons

In HT22 neurons, glucose uptake at 15 minutes increased significantly following AICAR treatment under sodium-containing conditions (Figure 2A). Co-treatment with phlorizin attenuated this increase, indicating sensitivity to sodium-dependent transport inhibition. Removal of sodium reduced basal glucose uptake and blunted AICAR responsiveness, with no significant enhancement observed under KRH − Na conditions. These results indicate that glucose uptake in HT22 neurons includes a sodium-sensitive component that is enhanced by AMPK activation.

Fructose uptake in HT22 neurons demonstrated a stronger dependence on sodium availability (Figure 2B). Under KRH+Na conditions, AICAR significantly increased fructose uptake despite some variability among biological replicates, and this effect was markedly reduced by phlorizin. In contrast, removal of sodium significantly attenuated the AICAR-induced increase in fructose uptake, and neither AICAR nor phlorizin significantly altered uptake under KRH − Na conditions. These findings indicate that fructose uptake in HT22 neurons is largely mediated by sodium-sensitive transport mechanisms and is responsive to metabolic activation.

#### BV2 Microglia

In BV2 microglial cells, glucose uptake at 15 minutes was significantly enhanced by AICAR under sodium-containing conditions, and this effect was attenuated by phlorizin (Figure 3A). Sodium removal had only a modest effect on basal glucose uptake but reduced the magnitude of the AICAR-induced increase, supporting a contribution of sodium-sensitive transport pathways to glucose handling in BV2 cells.

Fructose uptake in BV2 microglia displayed a distinct pattern compared to HT22 neurons (Figure 3B). Under KRH+Na conditions, AICAR significantly increased fructose uptake, and phlorizin partially reduced this effect. However, under sodium-free conditions, AICAR continued to enhance fructose uptake, and the inhibitory effect of phlorizin was reduced. These data suggest that fructose transport in BV2 microglia involves both sodium-dependent and sodium-independent components.

Collectively, these findings reveal cell-type-specific differences in hexose handling. HT22 neurons exhibit strong sodium sensitivity for fructose uptake, whereas BV2 microglia retains a sodium-independent component of fructose transport. Glucose uptake in both cell types is modulated by AMPK activation and is partially sensitive to sodium-dependent inhibition.

### Fructose Modulates Transporter Expression in HT22 Neurons and BV2 Microglia

To explore whether differences in hexose uptake were associated with changes in transporter expression, we examined mRNA levels of facilitated (GLUT family) and sodium-dependent (SGLT family) transporters in HT22 neurons and BV2 microglia following exposure to glucose or fructose.

#### HT22 Neurons

**Figure 4.**
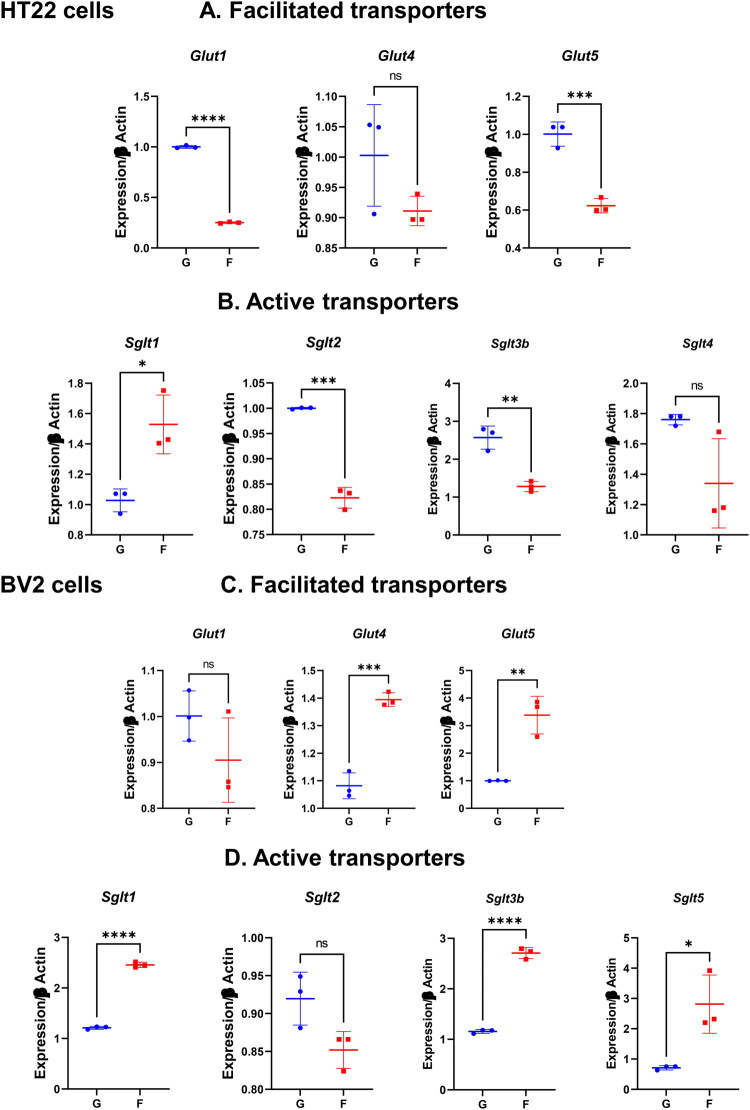
Fructose differentially regulates hexose transporter expression in neurons and microglia. (A and B) Expression of facilitated (GLUTs) and sodium-dependent (SGLTs) transporters in HT22 neurons following glucose or fructose exposure. Fructose suppresses Glut1 and Glut5 while inducing Sglt1 expression. (C and D) Transporter expression in BV2 microglia showing coordinated upregulation of Glut4, Glut5, and SGLT isoforms (Sglt1, Sglt3b, and Sglt5) in response to fructose. Data are presented as mean ± SD (*n* = 3 biological replicates per group). Statistical analysis was performed using one-way or two-way ANOVA followed by Tukey’s multiple-comparisons test; *P < 0.05; **P < 0.01*, P* < 0.01; ***P < 0.001; ****P < 0.0001.

In HT22 neurons, fructose exposure was associated with reduced expression of the facilitated transporters Glut1 and Glut5, while Glut4 expression remained unchanged (Figure 4A). Among sodium-dependent transporters, fructose increased Sglt1 expression but decreased Sglt2 and Sglt3b, whereas Sglt4 expression was not significantly altered (Figure 4B). These findings indicate a selective remodeling of both facilitated and sodium-dependent transporter expression in HT22 neurons in response to fructose.

#### BV2 Microglia

In contrast, BV2 microglia displayed a distinct expression pattern. Fructose treatment increased expression of the facilitated transporters Glut4 and Glut5, while Glut1 remained unchanged (Figure 4C). Within the sodium-dependent transporter family, fructose increased expression of Sglt1, Sglt3b, and Sglt5, whereas Sglt2 expression was reduced (Figure 4D).

Collectively, these results demonstrate cell-type–specific transcriptional responses of hexose transporters to fructose exposure, with distinct patterns observed between neuronal and microglial cells.

### Fructose Differentially Modulates Membrane Potential in BV2 Microglia and HT22 Neurons

To determine whether sodium-dependent fructose uptake directly alters membrane potential, we examined the effects of fructose on membrane potential in BV2 microglia and HT22 hippocampal neurons. Cells were loaded with the potentiometric dye DiBAC4 (3) (Ni et al., 2025), a negatively charged oxonol probe that accumulates in depolarized membranes, resulting in increased fluorescence, whereas reduced fluorescence reflects membrane hyperpolarization.

#### BV2 Microglia

**Figure 5.**
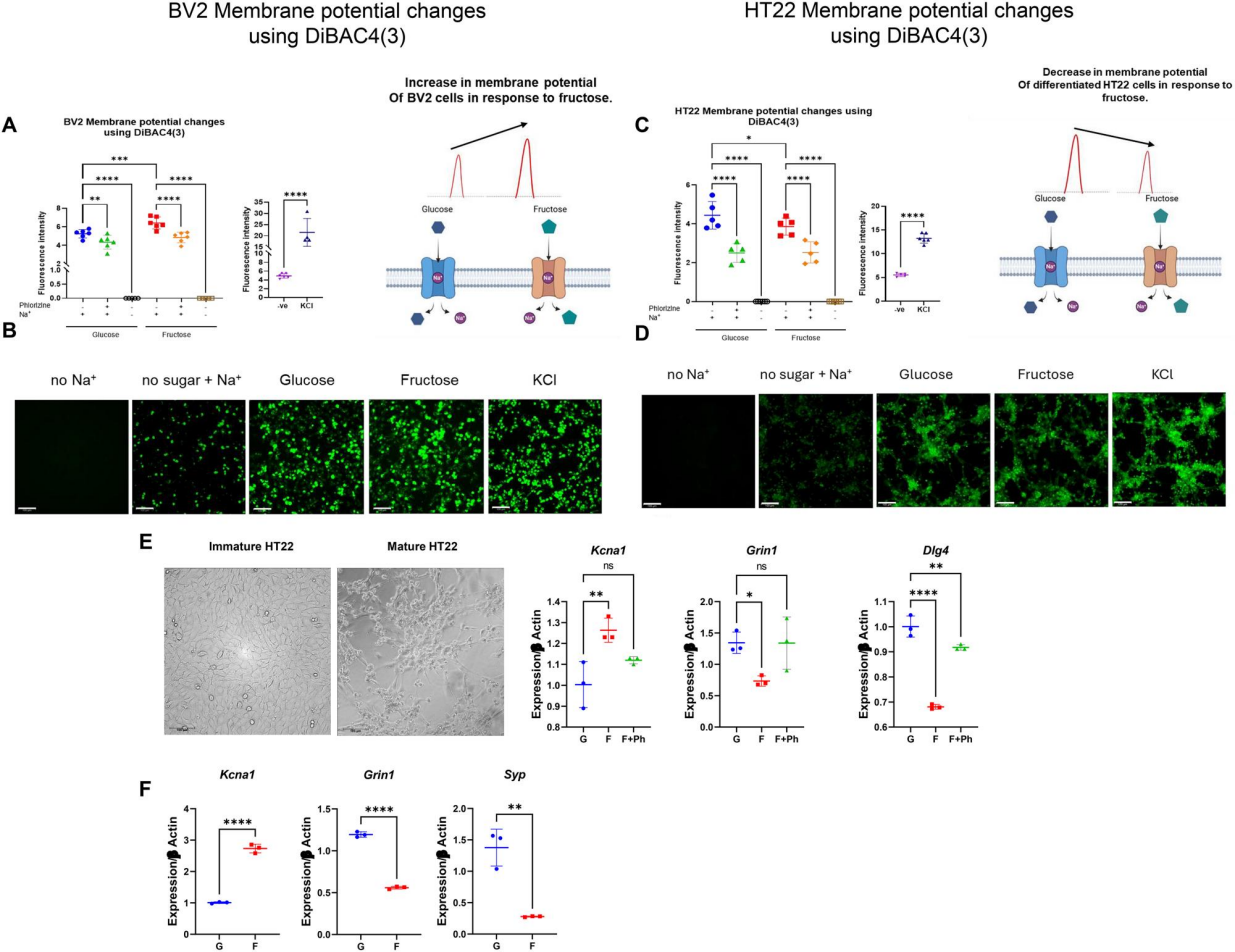
Fructose induces opposite membrane potential responses in BV2 microglia and HT22 neurons. (A and B) DiBAC4(3) fluorescence in BV2 microglia indicates fructose-induced membrane depolarization, attenuated by phlorizin. (C and D) HT22 neurons exhibit reduced DiBAC4(3) fluorescence following fructose exposure, consistent with membrane hyperpolarization, further enhanced by phlorizin. (E) Expression of the voltage-gated potassium channel Kcna1 in HT22 neurons following fructose exposure and phlorizin treatment. (F) Human iPSC-derived hippocampal neurons show increased Kcna1 expression and reduced synaptic markers (Syp, Dlg4) following fructose exposure. Data are presented as mean ± SD (BV2: *n* = 6; HT22: *n* = 5 biological replicates). Statistical analysis was performed using one-way or two-way ANOVA followed by Tukey’s multiple-comparisons test; *P < 0.05; **P < 0.01 *P* < 0.01; ***P < 0.001; ****P < 0.0001.

In BV2 microglia, fructose exposure produced a significant increase in DiBAC4 (3) fluorescence compared with glucose-treated cells, indicating membrane depolarization (Figure 5A). Co-treatment with phlorizin attenuated this fructose-induced increase, suggesting that sodium-sensitive transport contributes to the depolarizing response. Representative fluorescence images confirmed increased DiBAC signal following fructose treatment related to control and glucose conditions (Figure 5B).

#### HT22 Neurons

In contrast, HT22 neurons exhibited a decrease in DiBAC4 (3) fluorescence following fructose exposure relative to glucose-treated cells, consistent with membrane hyperpolarization (Figure 5C). Phlorizin treatment further enhanced the reduction in fluorescence under fructose conditions. Representative imaging supported the quantitative measurements (Figure 5D).

These findings indicate opposing membrane potential responses to fructose in microglial and neuronal cells.

#### Potassium Channel and Synaptic Gene Expression in HT22 Neurons

To explore potential mechanisms underlying fructose-induced membrane hyperpolarization in neurons, we examined the expression of potassium channels in HT22 cells under the same experimental conditions. Fructose exposure significantly increased expression of Kcna1, a voltage-gated potassium channel (Wang et al., 2025; Zhang et al., 2025), and this induction was reversed by phlorizin treatment (Figure 5E). Consistent with these findings, human iPScs–derived neurons expressing hippocampal markers, including GRIN1(Yin et al., 2019), also displayed a significant increase in Kcna1 expression upon fructose exposure, confirming that this response is not restricted to mouse HT22 cells (Figure 5F).

Neuronal scaffold proteins such as synaptophysin (SYP) (Li et al., 2023) and PSD-95 (DLG4) (Bustos et al., 2017) are essential organizers of pre- and postsynaptic specializations that cluster neurotransmitter receptors, ion channels, and signaling proteins to support synapse formation and plasticity (Vessey & Karra, 2007). Fructose exposure reduced expression of Synaptophysin (Syp), a key protein required for synapse formation (Li et al., 2023), and this effect phenocopied the reduction observed for Dlg4 (Psd-95) (Bustos et al., 2017) in differentiated HT22 neurons (Figure 5E,F). These cell-type–specific effects of fructose on membrane potential suggest that sodium-dependent fructose uptake differentially programs downstream secretory responses, prompting us to next examine how fructose regulates extracellular vesicle release in microglia and hippocampal neurons.

### Fructose Uptake Regulates Extracellular Vesicle Release in HT22 Neurons and BV2 Microglia

Given the distinct effects of fructose on hexose uptake, transporter expression, and membrane potential, we next examined whether fructose exposure alters extracellular vesicle (EV) release in HT22 neurons and BV2 microglia.

#### BV2 Microglia

**Figure 6.**
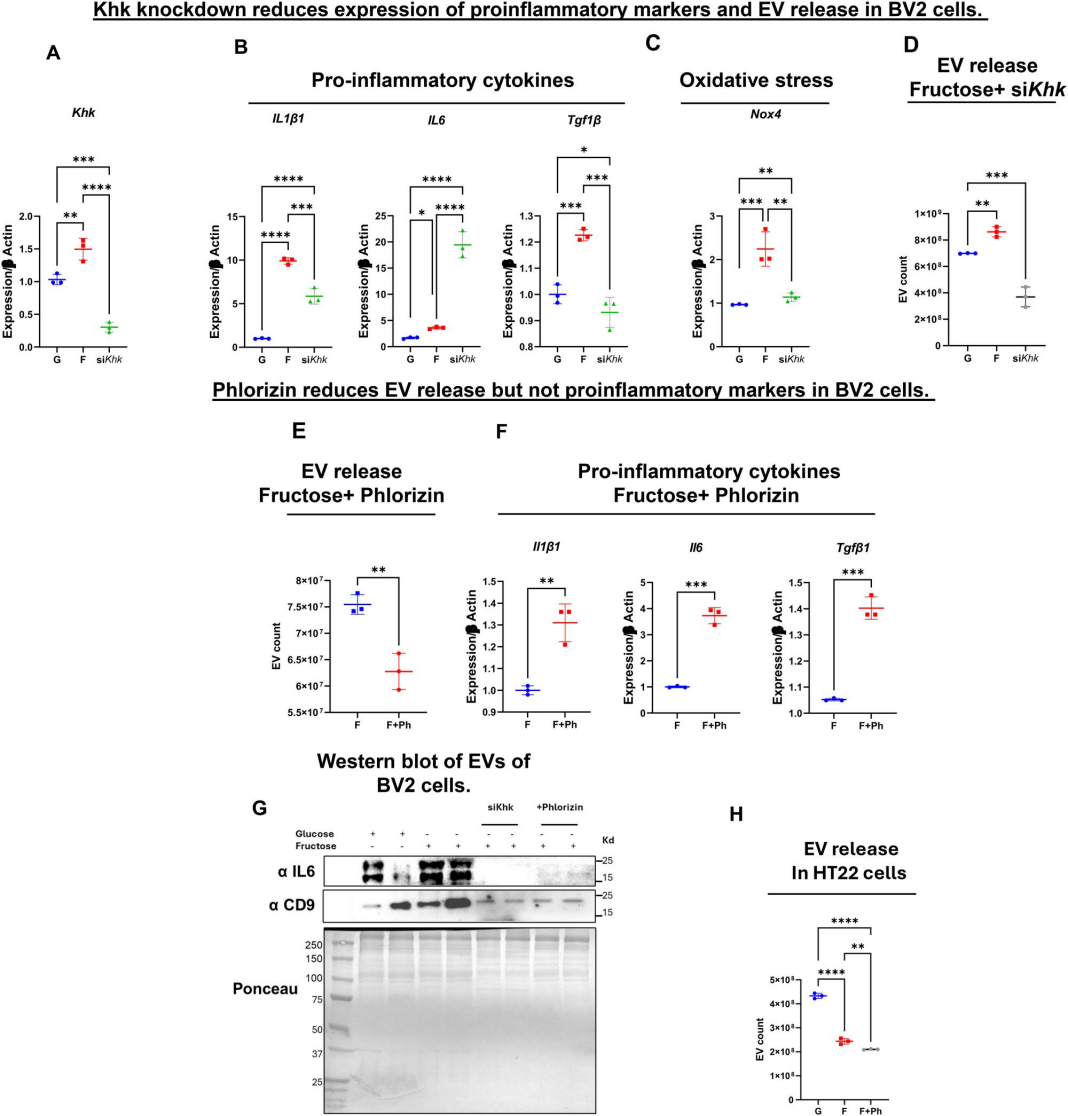
Sodium-dependent fructose uptake and fructolysis differentially regulate EV release in microglia and neurons. (A–C) Expression of Khk and inflammatory genes (Il1b, Il6, Tgfb1, and Nox4) in BV2 microglia following fructose exposure and Khk knockdown. (D) EV release from BV2 microglia, reduced following Khk silencing. (E and F) Effects of phlorizin on EV release and inflammatory gene expression. (G) Immunoblot of EV-associated proteins showing increased IL-6 following fructose exposure; CD9 used as an EV marker. (H) Fructose reduces EV release in HT22 neurons, further enhanced by phlorizin. Data are presented as mean ± SD (*n* = 3 biological replicates per group). Statistical analysis was performed using one-way or two-way ANOVA followed by Tukey’s multiple-comparisons test. *P < 0.05; **P < 0.01 *P* < 0.01; ***P < 0.001; ****P < 0.0001.

In BV2 microglia, fructose exposure significantly increased expression of Khk, confirming induction of fructose-metabolizing machinery (Figure 6A). Fructose also elevated pro-inflammatory cytokines (Il1b, Il6, Tgfb1) and the oxidative stress marker Nox4 (Figure 6B,C).

Silencing of Khk markedly reduced fructose-induced cytokine expression and oxidative stress markers. Importantly, KHK knockdown also significantly reduced fructose-induced EV release (Figure 6D), indicating that KHK activity contributes to both inflammatory gene induction and vesicle secretion under fructose exposure.

Pharmacological inhibition with phlorizin reduced fructose-induced EV release (Figure 6E). However, phlorizin did not suppress cytokine transcription; inflammatory gene expression remained elevated under fructose conditions (Figure 6F). Nanoparticle tracking analysis (NTA) confirmed that the isolated particles were predominantly within the expected extracellular vesicle size range and corroborated the observed treatment-dependent changes in EV abundance (Supplementary Fig. S4-B). Western blot analysis of EV isolates confirmed increased IL-6 protein content following fructose exposure, which was reduced by either KHK knockdown or phlorizin treatment, while CD9 expression verified vesicular identity (Figure 6G). Full-length, uncropped western blot images with molecular weight markers are provided in the Supplementary Figs. 1 and 2.

These findings indicate that fructose-induced EV release in microglia is sensitive to both KHK knockdown and pharmacological inhibition of sodium-dependent glucose transport, whereas inflammatory gene induction is predominantly KHK-dependent.

#### HT22 Neurons

In contrast to microglia, HT22 neurons exhibited a reduction in EV release following fructose exposure relative to glucose-treated cells (Figure 6H). NTA similarly confirmed the presence of extracellular vesicles within the expected size range and supported the observed reduction in EV abundance following fructose exposure (Supplementary Fig. S4-A).

Phlorizin treatment further reduced EV release under fructose conditions. Given the low baseline expression of Khk in HT22 cells, these results suggest that fructose influences EV secretion in neurons through mechanisms distinct from those observed in BV2 microglia.

Together, these data demonstrate divergent effects of fructose on EV release in neuronal and microglial cells. In BV2 microglia, fructose enhances EV secretion in a manner sensitive to both KHK knockdown and sodium-dependent transport inhibition. In contrast, fructose reduces EV release in HT22 neurons.

## Discussion

The brain exhibits remarkable metabolic plasticity by responding to fluctuations in its extracellular environment in a highly cell-type–specific manner (Domenico et al., 2025; Salmina, 2023). Under physiological conditions, brain cells rely primarily on glucose, with lactate serving as an important alternative energy substrate (Hyder, 2025). In contrast, excess fructose consumption and hyperglycemia-associated fructose production (Hwang et al., 2017) are increasingly linked to cognitive impairment and neuroinflammation (Celikbilek et al., 2018; Jiang et al., 2025; Rizzo et al., 2022; Zhang et al., 2025), yet the mechanisms by which fructose directly impacts brain cells remain poorly defined.

Here, we demonstrate that fructose elicits divergent responses in hippocampal neurons and microglia, characterized by differences in uptake dynamics, transporter expression remodeling, membrane potential regulation, and extracellular vesicle (EV) release. By integrating in vivo transcriptomic context with in vitro mechanistic studies, we identify cell-type–specific fructose handling strategies that may differentially shape excitability and inflammatory signaling within the hippocampal microenvironment.

**Figure 7.**
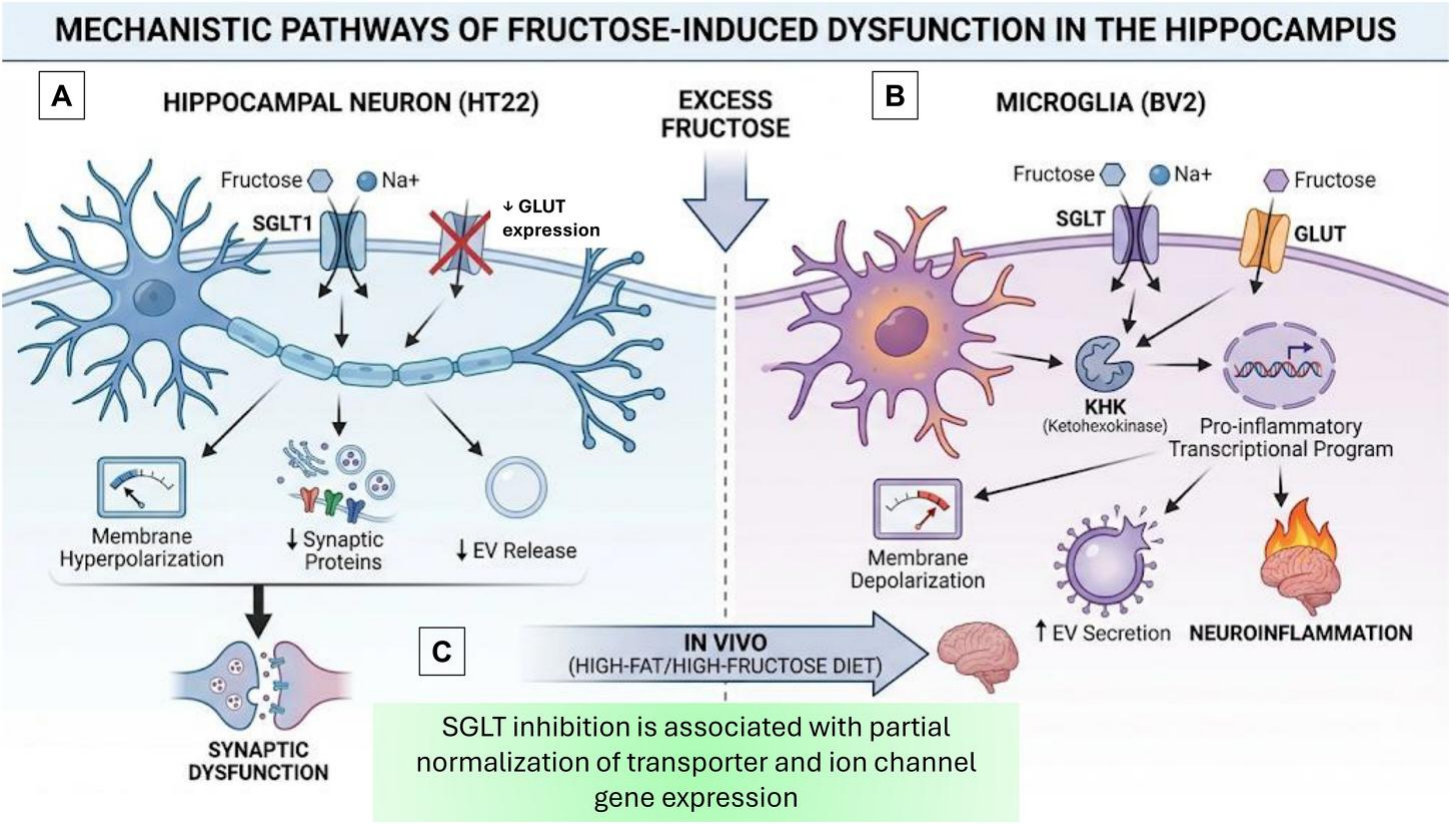
Divergent fructose handling governs hippocampal excitability and neuroinflammation. (A) Neuronal Metabolic Shunting: In HT22 hippocampal neurons, excess fructose exposure induces a compensatory shift in hexose transport, characterized by the upregulation of SGLT1 and a concomitant reduction in facilitated GLUT transporter expression. This Na^+^-coupled influx triggers membrane hyperpolarization and suppresses the expression of key synaptic proteins. Furthermore, fructose uptake attenuates extracellular vesicle (EV) biogenesis, collectively impairing neuronal connectivity and synaptic plasticity. (B) Microglial Inflammatory Priming: BV2 microglia exhibit high metabolic plasticity, utilizing a coordinated suite of SGLT and GLUT transporters to internalize fructose. This metabolic influx activates a ketohexokinase (KHK)-dependent transcriptional program, promoting a pro-inflammatory phenotype. Unlike neurons, microglial fructose metabolism drives membrane depolarization and robustly enhances EV secretion, potentially disseminating inflammatory signals throughout the hippocampal parenchyma. (C) Therapeutic Targeting of the SGLT Axis: In vivo administration of a high-fat/high-fructose (HFHF) diet leads to large-scale transcriptomic remodeling of ion channels and synaptic regulators. Pharmacological inhibition of SGLT activity (e.g., via phlorizin) effectively normalizes these pathological gene programs. This identifies SGLT-mediated fructose transport as a critical checkpoint and a novel therapeutic target for mitigating diet-induced cognitive decline and metabolic-related neurodegeneration.

GLUT5 (SLC2A5) is a fructose-selective transporter (Km approximately 6 mM) (Barron et al., 2016; Shepherd et al., 1992) and its expression in the brain is primarily associated with microglia rather than neurons (Li et al., 2023; Zhang et al., 2025). Basal fructose concentrations in the brain are generally low, and direct quantitative measurements across different brain regions under physiological and inflammatory conditions remain limited (Hwang et al., 2017). Accordingly, our findings in HT22 cells support a role for sodium-dependent fructose transport mechanisms without requiring substantial neuronal GLUT5 expression, whereas BV2 microglia retained both sodium-sensitive and sodium-independent components of uptake. The persistence of AICAR-stimulated glucose uptake under sodium-free conditions in HT22 cells suggests that AMPK activation may enhance both sodium-dependent and sodium-independent glucose transport pathways, consistent with a contribution from facilitated GLUT-mediated glucose uptake (Lemieux et al., 2003; Shrestha et al., 2021). We acknowledge that immortalized neuronal cell lines may not fully recapitulate the transporter profile of primary neurons in vivo. Consistent with emerging evidence that SGLTs are expressed in the central nervous system under metabolic stress (Yaribeygi et al., 2024; Yu et al., 2010), we observed coordinated regulation of SGLT family members in vitro and in hippocampal RNA-seq data. In vivo, phlorizin treatment was associated with partial normalization of transporter and ion channel gene expression, supporting the physiological relevance of sodium-dependent transport pathways (Figure 7C). Since the transcriptomic dataset reflects combined high-fat and fructose exposure in bulk tissue, these findings should be interpreted as contextual rather than proof of fructose-specific causality.

A key finding of this study is the striking divergence in neuronal and microglial responses to fructose. In neurons, fructose uptake was associated with membrane hyperpolarization, induction of voltage-gated potassium channels, suppression of synaptic proteins, and reduced extracellular vesicle (EV) release (Figure 7A). These changes are consistent with prior reports linking high-fructose diets to impaired hippocampal synaptic plasticity and cognitive function (Mazzoli et al., 2021; Wu et al., 2015) and suggest that restricted fructose transport capacity renders neurons vulnerable to fructose-induced functional suppression. Given the established role of neuronal EVs in synaptic maintenance and circuit communication (Zhang et al., 2024), reduced EV release may represent an additional mechanism through which fructose compromises neuronal integrity.

In contrast, microglia responded to fructose with membrane depolarization, induction of pro-inflammatory genes, and enhanced EV secretion (Figure 7B). Microglial EVs are increasingly recognized as vectors of inflammatory signaling within the brain microenvironment (Carata et al., 2024), and fructose-induced vesicle release may therefore amplify neuroimmune activation (Li et al., 2023; Shen et al., 2025; Wang et al., 2025). These functional effects were accompanied by broader remodeling of GLUT and SGLT transporter expression in microglia compared with neurons, consistent with greater metabolic flexibility.

The divergent responses observed in neurons and microglia may also have implications for neuron–glia metabolic crosstalk (Henn et al., 2022). Fructose exposure induced distinct effects on membrane potential and extracellular vesicle release in the two cell types, suggesting that differential transporter regulation and EV-mediated signaling may contribute to local metabolic and inflammatory communication within the hippocampal microenvironment. Further studies will be required to directly examine these interactions.

Our findings also distinguish transporter-dependent fructose entry from downstream fructolytic metabolism in microglia. Knockdown of ketohexokinase (KHK) attenuated both inflammatory gene induction and EV release, whereas inhibition of sodium-coupled fructose uptake with phlorizin selectively suppressed EV biogenesis without rescuing cytokine expression. These data suggest that KHK-mediated fructose metabolism plays a central role in inflammatory signaling, while transporter-dependent entry influences secretory output. Given that fructolysis bypasses key regulatory steps of glycolysis and has been linked to ATP depletion, oxidative stress, and endoplasmic reticulum stress (Elsaid et al., 2025; Flores Monar et al., 2025; Iizuka, 2023), KHK may represent a metabolic node connecting fructose exposure to inflammatory activation.

Fructose exposure in the brain arises not only from dietary intake but also from endogenous production via the polyol pathway during hyperglycemia (Hwang et al., 2017; Spinowitz & Altszuler, 1973). Notably, cerebrospinal fluid fructose concentrations have been reported to be approximately 20-fold higher than plasma fructose concentrations, reaching levels of approximately 0.1–0.2 mM despite circulating fructose concentrations in the low micromolar range (Hwang et al., 2017). These observations support local fructose generation within the central nervous system and suggest that sustained cerebral fructose stress may occur even in the absence of excessive dietary fructose. By identifying SGLTs and KHK as key regulators of fructose handling in the hippocampus, this study provides a mechanistic framework linking systemic metabolic dysregulation to alterations in excitability, vesicle signaling, and neuroimmune activation.

Some limitations warrant consideration. First, the in vivo transcriptomic analysis was performed on bulk hippocampal tissue and therefore does not provide cell-type-specific resolution, making it difficult to distinguish transcriptional regulation from potential changes in cellular composition. Second, the in vitro studies were intentionally designed to isolate fructose-specific mechanisms identified through hippocampal transcriptomic analysis and therefore do not fully reproduce the complex metabolic milieu associated with high-fat/high-fructose feeding in vivo. Furthermore, the use of immortalized neuronal and microglial cell lines may not fully recapitulate the physiological properties, transporter expression profiles, and cell–cell interactions present in primary brain cells and intact neuron–glia networks. Third, although phlorizin attenuated several fructose-induced responses, its lack of transporter specificity and limited translational applicability prevents definitive assignment of these effects to individual SGLT isoforms. Future studies using transporter-specific genetic and pharmacological approaches will be required to establish the contribution of individual sodium-dependent sugar transporters.

In summary, our findings reveal distinct fructose-handling strategies in hippocampal neurons and microglia and demonstrate that fructose transport and metabolism are associated with cell-type–specific changes in membrane polarization, extracellular vesicle release, and inflammatory signaling. These results extend current models of cerebral fructose metabolism and highlight transport- and metabolism-dependent pathways as potential modulators of hippocampal responses to metabolic stress.

## Supplementary Material

Supplemental Material
