# Supplementary material for: Hijacking Sodium–Glucose Cotransporters: Fructose Drives Neuronal and Microglial Dysfunction
Source: ASN Neuro. 2026 Jul 10;18(1):2696821. doi: 10.1080/17590914.2026.2696821 (PMC13360499; doi:10.1080/17590914.2026.2696821)
Supplement: Supplemental Material [file TASN_A_2696821_SM4998.pdf]

Supplementary Figure 1. Full-length immunoblot for IL-6 in extracellular vesicles.

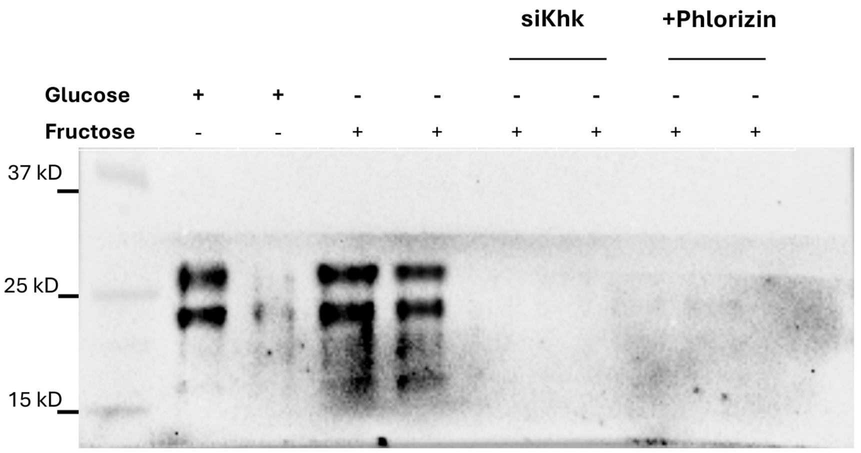

Supplementary Figure 1. Full-length immunoblot for IL-6 in extracellular vesicles.

Full-length, uncropped immunoblot showing IL-6 protein levels in extracellular vesicles (EVs) isolated from BV2 microglia under the indicated treatment conditions. Molecular weight markers are shown on the left. IL-6 expression increased following fructose exposure and was reduced by Khk knockdown or phlorizin treatment. Images were acquired under non-saturating conditions.

Supplementary Figure 2. Full-length immunoblot for CD9 as an extracellular vesicle marker.

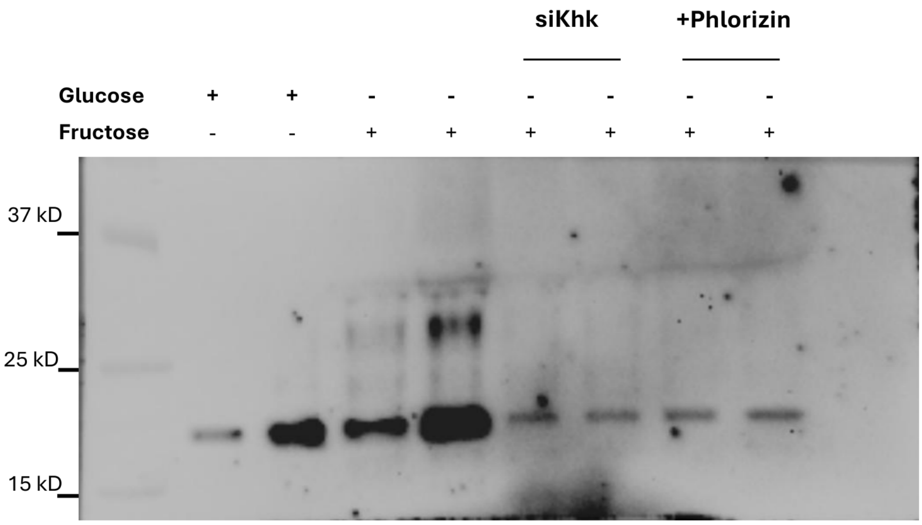

Supplementary Figure 2. Full-length immunoblot for CD9 as an extracellular vesicle marker.

Full-length, uncropped immunoblot showing CD9 expression in extracellular vesicles (EVs) isolated from BV2 microglia under the indicated treatment conditions. Molecular weight markers are shown on the left. CD9 was used as a canonical EV marker to confirm extracellular vesicle identity and consistent sample loading across treatment groups. Images were acquired under non-saturating conditions.

Supplementary Figure S3. High-fat/high-fructose diet induces remodeling of hippocampal GLUT and SGLT transporter expression.

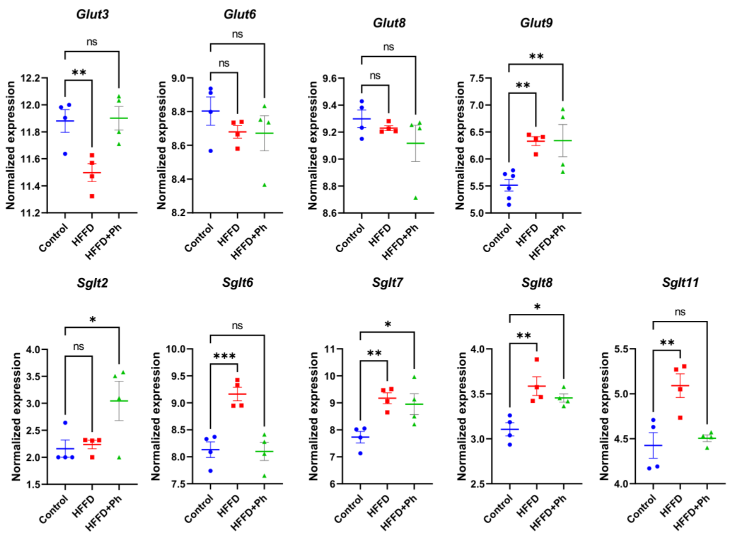

Supplementary Figure S3. High-fat/high-fructose diet induces remodeling of hippocampal GLUT and SGLT transporter expression.

RNA-seq analysis of facilitated glucose transporters (GLUTs) and sodium-dependent glucose transporters (SGLTs) in hippocampal tissue from control mice, mice fed a high-fat diet supplemented with 10% fructose (HFFD), and HFFD mice treated with phlorizin (HFFD+Ph). HFFD induced selective remodeling of both facilitated and sodium-dependent sugar transport pathways, including reduced GLUT3 expression and increased expression of GLUT9, SGLT6, SGLT7, SGLT8, and SGLT11. Several of these changes were partially normalized following phlorizin treatment. Data are presented as mean ± SD ($n = 4$ mice per group). Statistical analysis was performed using one-way ANOVA followed by Tukey's multiple-comparisons test. *$P < 0.05$, **$P < 0.01$, ***$P < 0.001$; ns, not significant.

Supplementary Figure S4. Characterization of extracellular vesicles by nanoparticle tracking analysis (NTA).

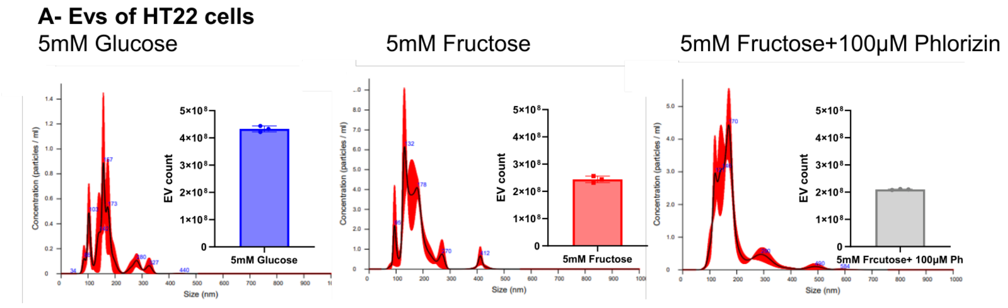

Supplementary Figure S4. Nanoparticle tracking analysis (NTA) of extracellular vesicles released from HT22 neurons and BV2 microglia.

(A) Representative NTA particle size-distribution profiles and quantification of extracellular vesicle (EV) concentration from conditioned media of HT22 cells treated with 5 mM glucose, 5 mM fructose, or 5 mM fructose plus 100 µM phlorizin. The majority of particles were distributed between approximately 80 and 250 nm, consistent with extracellular vesicle populations. Fructose treatment reduced EV release compared with glucose-treated controls, whereas phlorizin treatment further reduced EV abundance. Bar graphs show EV concentration (particles/mL) determined by NTA. Data are presented as mean ± SD with individual biological replicates shown (n = 3 independent experiments).

Supplementary Figure S4 (continue).

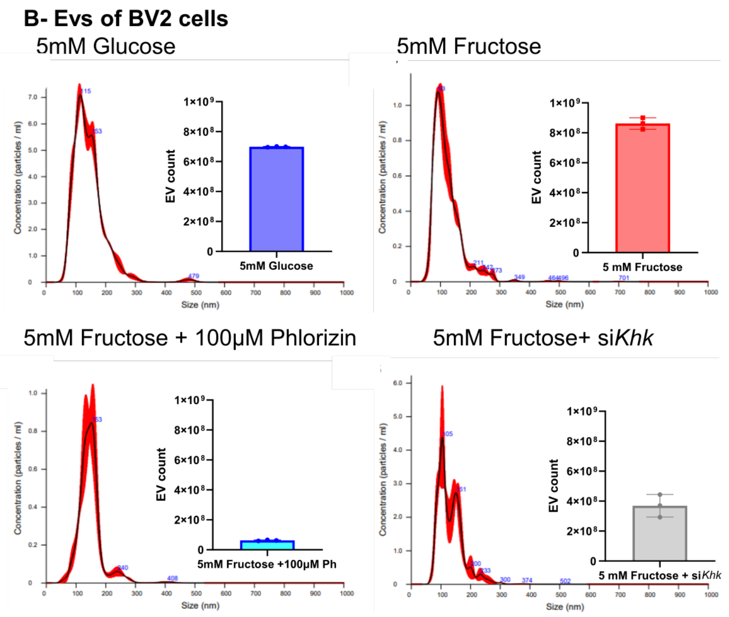

Supplementary Figure S4 (continued).

(B) Representative NTA particle size-distribution profiles and quantification of extracellular vesicle (EV) concentration from conditioned media of BV2 cells treated with 5 mM glucose, 5 mM fructose, 5 mM fructose plus 100 µM phlorizin, or 5 mM fructose following Khk knockdown (siKhk). The majority of particles were distributed between approximately 80 and 250 nm, consistent with extracellular vesicle populations. Fructose treatment increased EV release compared with glucose-treated controls, whereas phlorizin treatment and Khk knockdown reduced EV abundance. Bar graphs show EV concentration (particles/mL) determined by NTA. Data are presented as mean ± SD with individual biological replicates shown (n = 3 independent experiments.

Supplementary Figure S5. Immunofluorescence characterization of differentiated human iPSC-derived neurons.

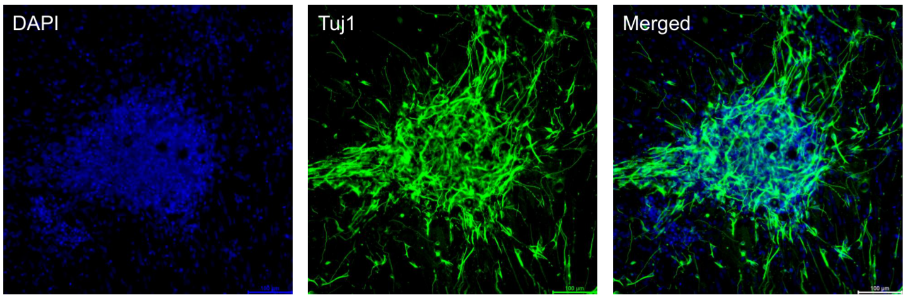

Supplementary Figure S5. Immunofluorescence characterization of differentiated human iPSC-derived neurons.

Representative immunofluorescence images of differentiated human induced pluripotent stem cell (iPSC)-derived neurons stained for the neuronal marker βIII-tubulin (TUJ1, green). Nuclei were counterstained with DAPI (blue). The merged image demonstrates successful neuronal differentiation, characterized by extensive TUJ1-positive neurite outgrowth and typical neuronal morphology. Scale bar = 100 µm.
